# 

**Published:** 1857-03

**Authors:** 


### CONTENTS.

### ORIGINAL COMMUNICATIONS.

PAGE

ARTICLE I.—The late John Newcom, M. D. By S. S. Satchwell, M. D., of New Hanover county, North Carolina,... 385

ARTICLE II.—Two cases of Typhoid Fever. By Wm. H. Philpot, M. D., of Redbone, Georgia,... 391

ARTICLE III.—Excerpts from the Case-Book of T. S. Powell, M. D., Sparta, Georgia,... 395

ARTICLE IV.—Remarks upon the Medical Properties and Therapeutical Application of Veratrum Viride. By V. H. Taliaferro, M. D., Atlanta, Georgia,... 399

### SELECTIONS.

A Monograph on Ovarian Tumors,... 407

The Relation of Chemistry to Practical Medicine,... 41

Aphorisms on the Hygiene and Nursing of Infants,... 419

### EDITORIAL AND MISCELLANEOUS.

Editorial Correspondence,... 432

University of Nashville,... 445

Prospectus of the North Carolina Journal of Medicine and Surgery,... 446

Invasion of the Toe-Nail,... 447

Dr. Arnott,... 448

Meteorological Observations for February, 1857, Atlanta, Ga.,... 448

